# Global and national burden of type 2 diabetes mellitus attributable to PM2.5 air pollution: An analysis of the GBD study from 1990 to 2019

**DOI:** 10.14814/phy2.70074

**Published:** 2024-10-07

**Authors:** Manan Raina, Pedro Salerno, Kush Doshi, Jieji Hu, Sanjay Rajagopalan

**Affiliations:** ^1^ Hawken High School Ohio USA; ^2^ Harrington Heart and Vascular Institute University Hospitals Cleveland Medical Center Cleveland Ohio USA; ^3^ Akron Nephrology Associates/Cleveland Clinic Akron General Medical Center Akron Ohio USA; ^4^ College of Medicine Northeast Ohio Medical University Rootstown Ohio USA; ^5^ School of Medicine Case Western Reserve University Cleveland Ohio USA

**Keywords:** air pollution, diabetes, particulate matter

## Abstract

Epidemiological studies have established a link between air pollution and an elevated risk of type 2 diabetes mellitus (T2DM). This study aims to measure the impact of T2DM related to fine particulate matter (PM2.5) pollution by examining death rates and disability‐adjusted life years (DALYs) from 1990 to 2019 in the United States of America. Using data from the Global Burden of Disease (GBD) database, we examined death and DALY rates per 100,000 populations in T2DM patients, specifically focusing on ambient particulate matter pollution (APMP) and household air pollution (HAP). We assessed average annual percentage change (AAPC) across various age and gender groups, states, and socio‐demographic index (SDI) categories. Our findings reveal a significant decline in death rates and DALYs in the United States of America over the last 30 years, with more pronounced decreases among females and older adults. Despite national progress, state‐level variations indicate complex interactions between environmental regulations, healthcare access, and socio‐economic factors. Some states, such as Oregon, Idaho, and Alaska, exhibited increased AAPC. Our study emphasizes the need for targeted policies and interventions to reduce PM_2.5_ exposure and further address regional disparities, ensuring continued improvement in public health outcomes.

## 1 INTRODUCTION

In 2019, air pollution was identified as one of the leading risk factors for disability‐adjusted life years (DALYs) and all‐cause mortality worldwide (Murray et al., 2020). Air pollution can be characterized by particulate matter (PM), categorized into PM_10_ and PM_2.5_. PM_10_ includes larger particles from fossil fuel combustion, construction sites, and unpaved roads that can reach the lungs but are typically trapped in the upper respiratory tract (Brown et al., 1950). However, epidemiological evidence has robustly linked exposure to fine particulate matter (PM2.5), with an aerodynamic diameter ≤2.5 μm, to a spectrum of adverse health outcomes, including cardiovascular, respiratory, and premature death (Thangavel et al., 2022). PM2.5 air pollutants can enter the respiratory tract and bloodstream, triggering biological responses such as endothelial dysfunction, immune response alterations, and endoplasmic reticulum stress (Thangavel et al., 2022). The Global Burden of Disease (GBD) Study 2019 has estimated that one‐fifth of the global burden of type 2 diabetes mellitus (T2DM) is attributable to chronic exposure to both ambient particulate matter pollution (APMP) and household air pollution from solid fuels (HAP) (Burkart et al., 2022). T2DM accounts for over 90% of all diabetes cases and is one of the most prevalent chronic diseases in the United States, imposing a significant burden on both the quality of life of individuals and the national economy (Vos et al., 2020). Assessing varying sources of air pollution exposure is crucial to developing effective and targeted health policies and interventions globally. This is particularly important in a diverse country such as the United States, where environmental and health policies can vary significantly from one state to another (Bowe et al., 2019). Variations in PM2.5 exposure levels, shifts in mortality rates, alterations in population age distribution, and socio‐environmental changes such as urbanization, population growth, and vegetation greenness influence the observed trends in PM2.5‐associated diabetes mortality (Bowe et al., 2019; Li et al., 2023). Current literature has primarily concentrated on the overall effects of PM2.5, with limited studies exploring the specific burden of APMP and HAP exposure on T2DM (Yang et al., 2020). This study examines the global and US health burden of T2DM attributed to varying PM2.5 air pollution levels via death rates and DALYs from 1990 to 2019.

## 2 METHODS

The GBD study contains the most extensive global epidemiological research to date. It assesses the disease burden from 286 causes of death, 369 diseases and injuries, and 87 risk factors across 204 countries and territories. These risk factors encompass metabolic, behavioral, and environmental aspects, including air pollution. The GBD 2019 study quantified the burden of T2DM attributable to APMP using the population‐attributable fraction method, which estimates the proportion of disease DALYs or mortality linked to a specific risk factor based on age, sex, location, and year. The population‐attributable fraction for T2DM due to APMP and HAP was calculated by integrating relative risk estimates from systematic reviews of epidemiological studies on APMP and HAP exposure levels (Murray et al., 2020). In the GBD database, APMP was defined as the population‐weighted annual average mass concentration of particles with an aerodynamic diameter <2.5 μm in a cubic meter of air (GBD 2019 Diseases and Injuries Collaborators, 2020). APMP was calculated using data from satellite‐based measurements of aerosol optical depth, ground measurements from 18,406 monitoring stations across 120 countries, and chemical transport model simulations (Shaddick et al., 2018). HAP was estimated using two components. The first component estimated the proportion of households using solid cooking fuels (coal, wood, charcoal, dung, and agricultural residues) from sources such as the World Health Organization Energy Database (Vos et al., 2020). The second component estimated the PM_2.5_ exposure level, which was estimated and extrapolated from studies measuring PM2.5 levels among individuals who used solid fuels for cooking (Bennitt et al., 2021). Varying solid fuels generate differing PM2.5 levels, which were extrapolated into population level data (Shupler et al., 2018). The GBD database was utilized to collect death rates and DALYs per 100,000 people in T2DM patients associated with PM2.5 air pollution, including APMP and HAP, from 1990 to 2019. DALYs were calculated as the sum of years of life lost and years lived with disability, which were calculated per GBD location (Institue for Health Metrics and Evaluation, 2024). In our study, we use the term “air pollution” to collectively refer to both APMP and HAP. The study analyzed the average annual percentage change (AAPC) and 95% confidence intervals for age and gender groups in the United States, including its 50 states and Washington DC, provided by the Institute for Health Metrics and Evaluation. The AAPC across the global socio‐demographic index (SDI) was also reviewed. SDI captures the socio‐economic development of geographic or administrative areas by combining information on the economy, education, and fertility rates. SDI is calculated as the geometric mean of indices including lag‐distributed per capita income, mean education for individuals aged 15 and older, and total fertility rate for those under 25. SDI is divided into high, high‐middle, middle, low‐middle, and low SDI categories. Statistical calculations were performed using the SPSS statistical package (version 21).

## 3 RESULTS

### 3.1 Death rates due to T2DM attributable to air pollution

In the United States, the overall death rates per 100,000 people in T2DM patients attributable to air pollution showed a decreasing trend between 1990 and 2019 [AAPC: −1.52% (95% CI −1.65 to −1.45) for APMP and − 2.39% (95% CI −3.83 to −1.74) for HAP; Table 1]. In the United States, females had a greater decrease in overall death rates compared with males for both APMP (AAPC of death rates −2.19% vs. −0.78%) and HAP (AAPC of death rates −2.91% vs. −1.47%). The 55+ year age group also had a greater decrease in overall death rates compared with the 20–54 year age group for both APMP (AAPC of death rates −2.55% vs. −1.51%) and HAP (AAPC of death rates −3.41% vs. −2.31%).

**TABLE 1:** AAPC over 1990–2019 in DALYs and death rate per 100,000 populations in T2DM attributable to APMP and HAP in the United States and stratification by age, gender, and global SDI.

	Death rate (95% CI)		DALYs rate (95% CI)	
	Ambient particulate matter pollution	Household air pollution from solid fuels	Ambient particulate matter pollution	Household air pollution from solid fuels
	AAPC (95% CI)	AAPC (95% CI)	AAPC (95% CI)	AAPC (95% CI)
United States of America	−1.52% (1.65% to −1.45%)	−2.39% (−3.83% to −1.74%)	−0.61% (−0.77% to 0.11%)	−1.51% (−2.91% to −0.78%)
Stratified by gender				
Female	−2.19% (−2.53% to −2.10%)	−2.91% (−4.18% to −2.28%)	−1.08% (−0.34% to −0.99%)	−1.88% (−3.17% to −1.14%)
Male	−0.78% (−1.09% to −0.70%)	−1.47% (−2.85% to −0.74%)	−0.17% (0.54% to −0.14%)	−0.95% (−2.38% to −0.10%)
Stratified by age groups				
<5 years	Data not available			
5–19 years				
20–54 years	−1.51% (−1.85% to −1.44%)	−2.31% (−3.71% to −1.68%)	−0.81% (−1.12% to −0.77%)	−1.67% (−2.92% to −0.84%)
55+ years	−2.55% (−2.88% to −2.47%)	−3.41% (−4.83% to −2.75%)	−1.54% (−1.81% to −1.48%)	−2.44% (−3.82% to −1.71%)
Stratified by global SDI				
High SDI	−0.35% (−0.38% to −0.32%)	−6.07% (−8.41% to −4.47%)	0.70% (0.78% to 0.62%)	−5.90% (−8.19% to −4.38%)
High‐middle SDI	2.36% (2.17% to 2.55%)	−2.88% (−4.04% to −2.14%)	2.84% (3.02% to 2.72%)	−2.96% (−4.25% to −2.14%)
Middle SDI	4.73% (4.24% to 5.21%)	−1.02% (−1.68% to −0.60%)	4.86% (5.40% to 4.48%)	−1.29% (−1.99% to −0.88%)
Low‐middle SDI	6.18% (5.14% to 7.68%)	0.03% (−0.49% to 0.04%)	6.24% (7.68% to 5.24%)	0.06% (−0.53% to 0.08%)
Low SDI	3.74% (2.67% to 5.93%)	−0.95% (−1.74% to 0.62%)	4.13% (6.29% to 2.98%)	−0.63% (−1.56% to −0.45%)

**FIGURE 1:**
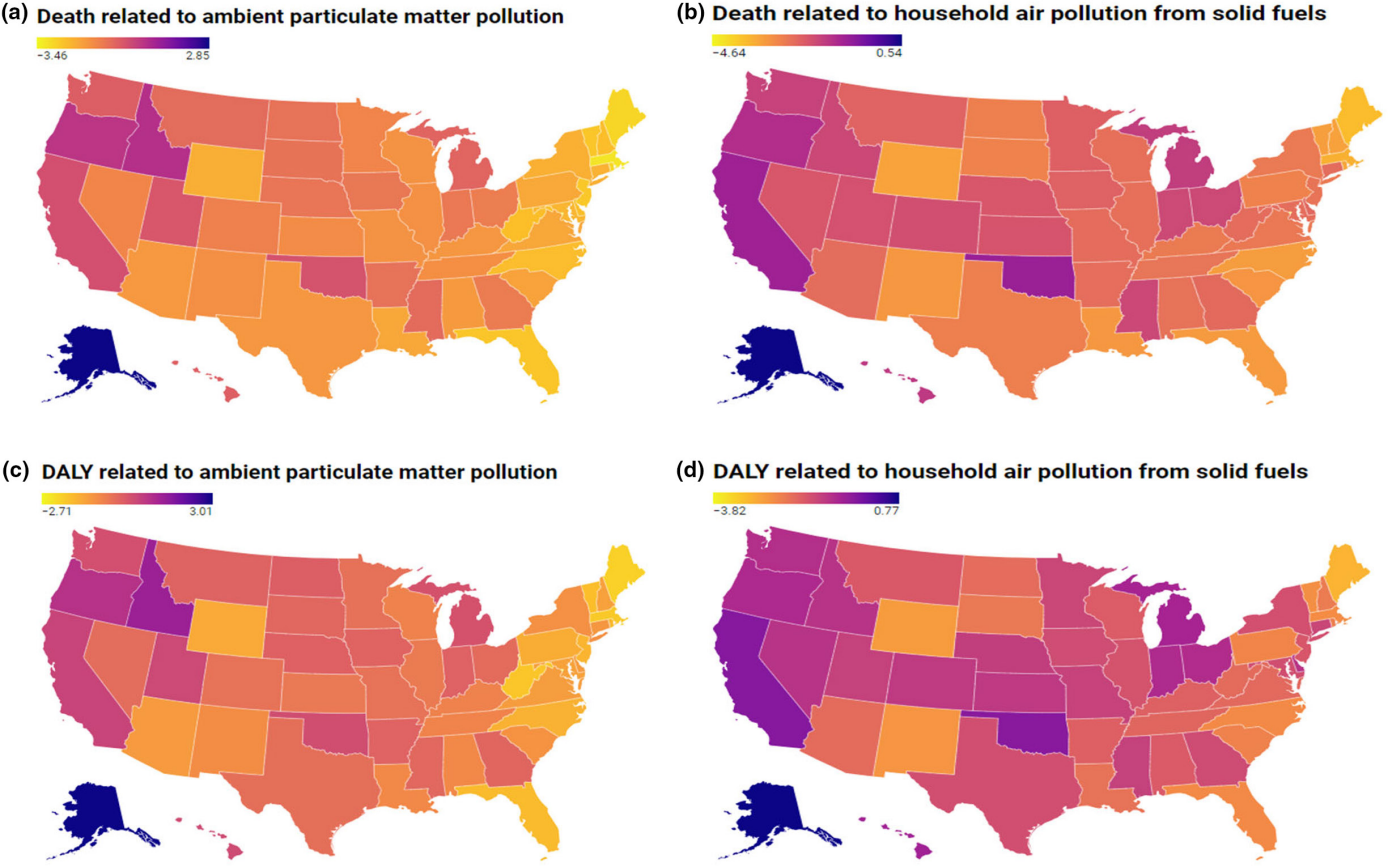
AAPC over 1990–2019 in DR and DALYs per 100,000 populations in T2DM attributable to APMP and HAP across different US states and Washington DC. Data obtained from the GBD database (Institute for Health Metrics and Evaluation, 2024). (a) There was an overall decrease in death rates from T2DM attributable to APMP, except in 3 states: Oregon, Idaho, and Alaska, with Alaska having the greatest increase from 1990 to 2019. (b) There was an overall decrease in death rates from T2DM attributable to HAP, except in Alaska, where it increased 0.54% from 1990 to 2019. (c) There was an overall decrease in DALYs from T2DM attributable to APMP, except in 10 states, with Alaska having the greatest increase from 1990 to 2019. (d) There was an overall decrease in DALYs from T2DM attributable to HAP, except in Alaska, where it increased 0.77% from 1990 to 2019. APMP, ambient particulate matter pollution; DALYs, disability adjusted life years; HAP, household air pollution; T2DM: Type 2 diabetes.

Across the different US states, the decrease in death rates for T2DM attributable to APMP and HAP varied. For APMP, there was a greater decrease in death rates across 22 states including Washington, DC (AAPC of death rates −3.46% to −1.59%). In 25 states, there was a smaller decline in death rates due to APMP (AAPC of death rates −1.51% to −0.14%). However, in three states, Oregon (AAPC: +0.44%), Idaho (AAPC: +0.55%), and Alaska (AAPC: +2.85%), there was an increase in death rates due to APMP (Figure 1a).

For HAP, 29 states, including Washington, DC, had a greater decrease in death rates (AAPC of death rates due to HAP −4.64% to −2.41%). In 20 states, there was a smaller decline in death rates due to HAP (ranging from −2.37% to −0.94%). However, an increase in death rates due to HAP was observed for Alaska (AAPC of death rates: +0.54%) (Figure 1b).

Death rates for T2DM attributable to APMP decreased for the high SDI index but increased for the other SDI indexes. Death rates for T2DM attributable to HAP either decreased or remained flat across the different SDI indices (Table 1).

### 3.2 DALYs due to T2DM attributable to air pollution

In the United States, the overall DALY rates per 100,000 people due to T2DM attributable to air pollution showed a decreasing trend between 1990 and 2019 [AAPC of DALYs −0.61% (95% CI −0.77 to 0.11) for APMP and −1.51% (95% CI −2.91 to −0.78) for HAP; Table 1]. In the United States, females had a greater decrease in overall DALYs compared with males for both APMP (AAPC of DALYs −1.08% vs. −0.17%) and HAP (AAPC of DALYs −1.88% vs. −0.95%) (Table 1). The 55+ years age group had a greater decline in DALYs compared to the 20–54 years age group for both APMP (AAPC of DALYs −1.54% vs. −0.81%) and HAP (AAPC of DALYs −2.44% vs. −1.67%).

Across the different US states, the decrease in the DALYs for T2DM attributable to APMP and HAP varied. For APMP, there was a greater decrease in DALYs in 24 states, including Washington, DC (AAPC of DALYs −2.71% to −0.65%). In 16 states there was a smaller decline in DALYs attributable to APMP (AAPC of DALYs −0.51% to −0.04%). However, there was an increase in DALYs attributable to APMP in 10 states (AAPC of DALYs +0.02% to +3.01%) (Figure 1c).

For HAP, there was a greater decrease in DALYs in 24 states including Washington, DC (AAPC of DALYs −3.82% to −1.51%). In 25 states, there was a smaller decline in DALYs attributable to HAP(AAPCof DALYs −1.45% to −0.31). However, there was an increase in DALYs in Alaska (AAPC of DALYs: +0.77%) (Figure 1d).

A diverse trend was observed concerning DALYs for T2DM in each SDI index. The AAPC of DALYs for T2DM attributable to HAP decreased or remained stable. The AAPC of DALYs for T2DM attributable to APMP increased across the different SDI indices (Table 1).

## 4 DISCUSSION

This report demonstrates a notable decline in death rates and DALYs from T2DM attributable to air pollution in the United States from 1990 to 2019. The national trend shows a 33% reduction in PM2.5 concentrations since 2000 and a nearly 50% drop in overall all‐cause mortality from PM2.5 exposure, highlighting substantial progress in air quality management. The Clean Air Act implemented by the Environmental Protection Agency has led to the strict enforcement of national air quality standards, effectively regulating the release of major air pollutants such as nitrogen oxides, PM2.5, and ozone, thereby promoting better environmental and public health (U.S. EPA, 2009). However, variability in the AAPC for death rates and DALYs across states indicates a complex interaction between environmental regulations, healthcare accessibility, demographic shifts, and socioeconomic factors. Specifically, we noted an increase in the AAPC for DALYs and death rates in Oregon, Idaho, and Alaska. These states likely encounter distinct environmental or policy challenges that offset the generally positive national trends. For instance, the American Lung Association's “State of the Air” 2024 report highlights cities in the above states as having some of the worst air pollution in the nation (American Lung Association, 2024). This may be due to factors such as wood‐burning stoves and wildfire smoke. However, other states such as California and Washington still had a decrease in the AAPC for death rates and DALYs for T2DM attributable to air pollution despite having heavily polluted cities. This suggests the presence of localized environmental challenges that contrast with the positive trends observed in other regions (American Lung Association, 2020).

Regional and state‐level policies may also play a role. Examples of policies that would decrease APMP include incorporating person‐centric built environments, increasing urban green spaces and parks, and building vegetation buffers along highways (Yeager et al., 2020). Meteorological sources of APMP such as desert dust and forest fires should be optimized with strategies such as soil conservation, forestry, and prescribed burning (Riggs et al., 2018). On a personal levels, individuals should be encouraged to reducing their air pollution exposure from activities like cooking, home heating, and smoking, through personalized approaches like smoking cessation, low‐emission stoves, proper ventilation, and indoor air filters (Bhatnagar, 2022).

There was a greater reduction in both DALYs and death rates attributable to both HAP and APMP among females and older age groups, which correspond with previously published studies (Honda et al., 2017; Park et al., 2015). This suggests that these demographic groups not only experience different levels of air pollution and have varying underlying mortality rates but may exhibit greater sensitivity to air pollution exposure. Death rates and DALYs among females and the 55+ year group declined, consistent with the overall reduction in air pollution across the United States.

For APMP, the variations found in DALYs and death rates may result from persistent and rising ambient air pollution in urbanizing regions. Despite emission control efforts, APMP poses significant health risks, especially when regulatory and technological advancements lag behind increases in pollution. Prolonged exposure to APMP exacerbates chronic health conditions, increasing the overall health burden. Indeed, every 10‐μg/m^3^ increase in PM2.5 will lead to a 36% increase in diabetes‐related mortality (Huang et al., 2024).

In contrast, the decrease in death rates and DALYs for HAP from solid fuels is largely due to improved access to cleaner cooking and heating technologies and public health interventions. Programs replacing traditional solid fuels with cleaner alternatives like liquid petroleum gas or electricity have effectively reduced indoor air pollution. Nevertheless, disparities in socio‐economic conditions and racial inequalities continue to contribute independently to the health burdens associated with PM_2.5_ exposure (Bowe et al., 2019).

Globally, the majority of T2DM attributable to PM_2.5_ is driven by ambient air pollution, except in sub‐Saharan Africa, where household air pollution remains the primary contributor (Noubiap et al., 2015). Since 1990, this attributable burden has doubled, largely due to population growth and aging (Burkart et al., 2022). While reductions in the burden from household air pollution were achieved, they were largely counterbalanced by an increased burden from ambient PM2.5 exposure. A majority of the attributable burden occurred in Asia, sub‐Saharan Africa, and South America (Shupler et al., 2018). These findings emphasize the need for targeted research and tailored policy to effectively reduce ambient PM2.5 levels and address the disparities across different populations and regions.

## 5 LIMITATIONS

There are several limitations to our study. The GBD dataset likely does not address all confounding factors despite comprising of higher‐quality cohort and case–control studies. We attempted to account for socioeconomic status in our study by the inclusion of the SDI index, but other factors such as genetics, access to healthcare, dietary habits, and physical activity levels were not included in our study. Other forms of pollution, such as traffic, aquatic, and noise pollution were not included in our study. Finally, the GBD database has been refining air pollution calculations by including more data from governmental organizations and including data from an increasing number of satellites. Earlier estimates of air pollution may have suffered from underreporting and introduced inconsistencies in trend analyses over the 30 years of data included.

## 6 CONCLUSION

This study demonstrates a significant reduction in death rates and DALYs associated with T2DM attributable to air pollution in the United States from 1990 to 2019. The overall decline reflects an overall national decrease in PM2.5 concentrations, largely driven by regulatory measures such as the Clean Air Act. Notably, states like Oregon, Idaho, and Alaska had an increase in death rates and DALYs, which suggest that local and state level regulations and environmental events may counteract improvements seen on the national level. Additionally, the more pronounced reductions found in females and those over the age of 55 suggest that different demographics have varying degrees of sensitivity to air pollution levels. Socioeconomic disparities continue to influence healthcare outcomes, as seen in death rates in T2DM attributable to APMP. These findings emphasize the need for targeted research and the development of regional and state‐level policies to further reduce the burden of air pollution and ensure that the benefits of clean air are equitably distributed to all populations.

## FUNDING INFORMATION

No funding was received for this study.

## CONFLICT OF INTEREST STATEMENT

The authors declare no conflicts of interest.

## ETHICS STATEMENT

This work does not involve trials on any human or animals. Data supporting this study's findings are available upon reasonable request from the corresponding author.

## Data Availability

The datasets analyzed during the current study are available from the corresponding author upon reasonable request.
